# Web-based telemonitoring of visual function and self-reported postoperative outcomes in cataract care: international multicenter randomized controlled trial

**DOI:** 10.1097/j.jcrs.0000000000001492

**Published:** 2024-08-26

**Authors:** Janneau L.J. Claessens, Joukje C. Wanten, Noël J.C. Bauer, Rudy M.M.A. Nuijts, Violette Vrijman, Esen Selek, Rob J. Wouters, Nicolaas J. Reus, Fallon J.G.M. van Dorst, Oliver Findl, Manuel Ruiss, Karl Boden, Kai Januschowski, Saskia M. Imhof, Robert P.L. Wisse

**Affiliations:** From the Department of Ophthalmology, University Medical Centre Utrecht, Utrecht, the Netherlands (Claessens, Imhof, Wisse); University Eye Clinic Maastricht, Maastricht University Medical Center+, Maastricht, the Netherlands (Wanten, Bauer, Nuijts); Oogcentrum Noordholland, Heerhugowaard, the Netherlands (Vrijman, Selek, Wouters); Department of Ophthalmology, Amphia Hospital, Breda, the Netherlands (Reus, van Dorst); Vienna Institute for Research in Ocular Surgery, a Karl Landsteiner Institute, Hanusch Hospital, Vienna, Austria (Findl, Ruiss); Eye Clinic Sulzbach, Knappschaft Hospital Saar, Sulzbach, Germany (Boden); Mount Saint Peter Eye Clinic, Trier, Germany (Januschowski).

## Abstract

**Purpose:**

To compare web-based, self-administered follow-up after cataract surgery to conventional face-to-face follow-up.

**Setting:**

Eye clinics in the Netherlands, Austria, and Germany.

**Design:**

Randomized controlled trial with an embedded method comparison study (ClinicalTrials.gov: NCT04809402).

**Methods:**

Routine patients with cataract were randomized into 2 groups: The telemonitoring group undertook web-based vision self-assessments and questionnaires from home, while the usual care group received conventional care. All participants had a 4- to 6-week postoperative clinic visit for safety and validation purposes. Outcomes included, the web test's accuracy for assessing postoperative visual acuity (VA) and refractive error, adverse event rates, and patient-reported outcome measurements (PROMs).

**Results:**

94 participants (188 eyes) were enrolled. Web-based uncorrected distance VA testing demonstrated a negligible mean difference (−0.03 ± 0.14 logMAR) when compared with conventional Early Treatment Diabetic Retinopathy Study chart testing, with 95% limits of agreement ranging from −0.30 to 0.24 logMAR. The web-based refraction assessment overestimated the postoperative refractive error (mean difference in spherical equivalent 0.15 ± 0.67 diopters), resulting in a poorer corrected distance VA compared with subjective refraction (mean 0.1 vs −0.1 logMAR). Rates of adverse events and unscheduled consultations were minimal across both groups. Preoperative and postoperative PROM questionnaires had a 100% response rate. Visual functioning (Catquest-9SF and National Eye Institute Visual Function Questionnaire-25) improved postoperatively (mean improvement −0.80 and 16.70, respectively) and did not significantly differ between the 2 groups.

**Conclusions:**

The patients with cataract in this study effectively provided postoperative outcome data using a web interface. Both conventional and web-based follow-ups yielded similar PROMs and adverse event rates. Future developments should reduce the variability in the web-based VA test and yield representative refraction outcomes.

## Abstract 2

Cataract patients in this study effectively used a web-based platform to independently assess visual function and report postoperative outcomes, highlighting its potential for telemonitoring after cataract surgery.

Cataract surgery stands as the most performed surgical procedure across European member states, with demands continuing to rise in our aging society.^1^ Workloads for eyecare professionals are ever-increasing and jeopardize access to ophthalmic care. Solutions that increase efficiency and productivity are, therefore, desired. Optimization of staff time by telehealth and self-management can lead to productivity gains, as a greater volume of patients can be managed with similar resources.^2^ Other important advantages are related to the lower frequency of clinic visits and include reduction of travel-related expenditures, carbon dioxide emissions, and absenteeism from work.^3^

Although national protocols vary, conventional cataract surgery follow-up typically involves routine face-to-face examinations to assess postoperative visual function and detect adverse events.^4^ Advances in surgical techniques and routine use of intraocular antibiotics have greatly improved safety of this procedure, and most of the postoperative check-ups are uneventful.^5,6^ This renders cataract surgery follow-up a compelling field for using telehealth for remote care delivery.

This trial is the first to evaluate cataract surgery follow-up involving remote self-assessments of visual function and self-reported outcome measurements collected at home, compared with conventional clinical practice where those assessments are conducted at clinics and obtained by trained staff.

## METHODS

### Study Design

A detailed overview of the study design can be found in the published study protocol.^7^ This randomized controlled trial was performed at 6 eye clinics: 4 located in the Netherlands (University Medical Center Utrecht, Maastricht University Medical Center+, Amphia Hospital Breda, Oogcentrum Noordholland), 1 in Austria (Vienna Institute for Research in Ocular Surgery), and 1 in Germany (Augenklinik Sulzbach). The study was registered at ClinicalTrials.gov, number NCT04809402, and the protocol for this study was designed according to the SPIRIT 2013 guidelines.^8^ The study was approved by the Medisch Ethische Toetsingscommissie Utrecht, the Netherlands (NL74625.041.21); the Ethikkommission der Stadt Wien, Austria (EK 20-334-0121); and the Ethikkommission Saarbrücken, Germany (Ha 44/18). Written informed consent was obtained from all participants.

Between April 2021 and January 2023, consecutive patients planned for bilateral cataract surgery (either delayed sequential or immediate sequential) were invited to participate. Exclusion criteria were cataract surgeries combined with other procedures; presence of ocular comorbidities that negatively influence postoperative visual acuity (VA), such as amblyopia, age-related macular degeneration, diabetic retinopathy, glaucoma, or uveitis; an insufficient command of the Dutch, German, or English language; and not being able to access the web-based eye test (Easee BV, Amsterdam, the Netherlands) at home. The latter required access to a computer or tablet and a smartphone (possibly obtained by a relative) and an internet connection. Before enrollment, all interested patients were requested to try a demo version of the web-based eye test (a shortened test version). Those who were unable to access this demo were excluded from participation. Assistance by a relative was always recommended, though not strictly required.

**Figure 1.**
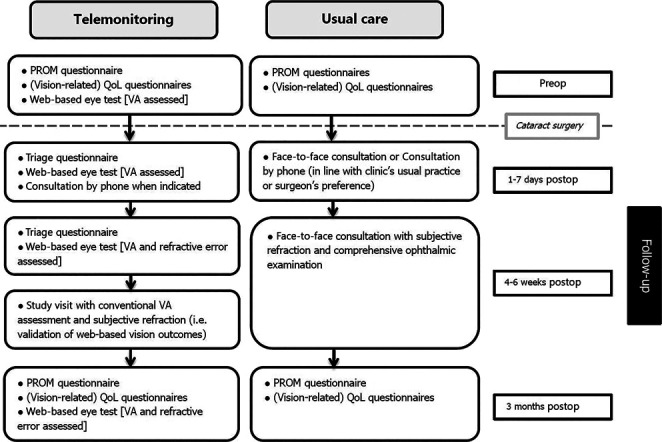
Study assessments. PROM = patient-reported outcome measurements, assessed by Catquest-9SF questionnaire (vision-related); QoL = (vision-related) quality of life, assessed by the National Eye Institute Visual Function Questionnaire-25 and EuroQol 5-Dimension 5-level questionnaire^14–16^

After enrollment, patients were randomly assigned (1:1) to 1 of the 2 follow-up groups, using a web-based system stratified by center and age (<69 and ≥69 years). Participants of the telemonitoring group performed postoperative self-assessments at home (remote vision self-assessments and questionnaires) while those of the usual care group had conventional postoperative consultations dictated by local preferences and regulations. The postoperative self-assessments were offered at 3 distinct points in time: an early assessment within 1 to 7 days postoperatively, a late assessment 4 to 6 weeks postoperatively, and a final assessment at 3 months postoperatively. All participants underwent a remote assessment of patient-reported outcome measurements pre and postoperatively, and all participants underwent an in-office clinical assessment 4 to 6 weeks postoperatively. The telemonitoring group was invited to perform the remote assessments within 5 days before the in-office assessment at 4 to 6 weeks postoperatively. As such, a method comparison study design was embedded in the telemonitoring arm, comparing the home-based self-assessment with the conventional in-office clinical assessment of VA and refractive error. An overview of the study assessments is presented in Figure 1. Invitations for questionnaires and/or web-based eye tests were sent through email. Any unexpected consultations or adverse events were recorded for both groups.

### Outcome Measures

#### Accuracy of the Web-Based Vision Self-Assessment (Telemonitoring Group Only)

**Figure 2.**
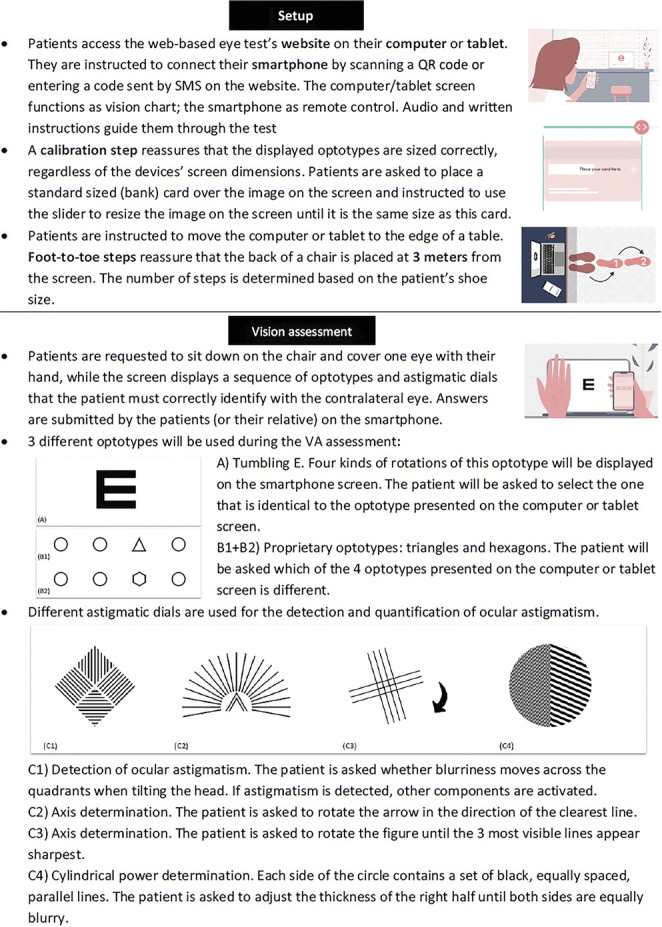
Overview of the web-based test flow. Collected parameters of the web-based test include CDVA/UDVA and refractive error (sphere, cylinder, axis).

The web-based eye test evaluated in this study allows self-assessments of visual function at home. Easee (Amsterdam, the Netherlands) uses an ISO 13485 Quality Measurement System, and the tool is classified as Conformité Européenne class 2A medical device according to the Medical Device Regulation 2017/745.^9^ An overview of the test flow and assessed determinants of visual function is presented in Figure 2.

The web-based test's accuracy was evaluated by comparing the home-assessed uncorrected distance VA and refraction to the outcomes obtained by trained staff during the validation visit at 4 to 6 weeks postoperatively. These assessments only refer to the telemonitoring group, and comparisons are made of data acquired in the same subject. Repeatability of the web-based test was evaluated by comparing the outcomes obtained 3 months postoperatively with those obtained 4 to 6 weeks postoperatively. Participants were asked whether they had experienced a change in vision before the start of the final eye test, and if so, these cases were excluded from the analysis. Based on studies on intraindividual variability in repeated VA and refraction assessments, differences between assessments up to 0.15 logMAR or 0.5 diopters (D) were considered normal measurement variation.^10–13^

The web-based VA assessment consists of 3 parts. In each part, a different optotype is used (Figure 2). This results in 3 individual scores that are averaged to calculate the final web-based VA score. A human over-read was performed after data collection. All web-based assessments were checked on individual levels to detect a within-test variation >0.2 logMAR between the 3 separate VA tests of 1 monocular assessment. As the true web-based test score cannot be reliably determined for these eyes, these were excluded from the comparisons and discussed separately. Conventional VA was assessed by Early Treatment Diabetic Retinopathy Study (ETDRS) charts at 4 meters (distance) and Sloan ETDRS charts at 40 centimeters (near).

The test's algorithm will interpret any VA poorer than −0.1 logMAR (ie, 1.25 Snellen decimal) to be caused by a refractive error. Astigmatic dials will determine the presence and magnitude of a cylinder. The type of refractive error (myopia or hyperopia) is based on an adapted red/green duochrome test combined with a short questionnaire. Algorithm updates had taken place after the start of the trial, resulting in a new method of calculating the spherical equivalent (SEQ). This updated algorithm performance is reported alongside the performance of the original algorithm. Subjective refraction was obtained at the clinic by trained optometrists masked for the web-based outcomes. After this assessment, corrected distance VA (CDVA) was measured using the prescription of both the subjective refraction and the web-based assessment.

#### Safety

For both study groups, all adverse events and unexpected consultations were registered. Participants of the telemonitoring group were requested to fill out a triaging questionnaire before each web-based vision assessment. Outcomes of these questionnaires were evaluated.

#### Self-Reported Outcome Measurements

Both study groups were sent digital questionnaires about (vision-related) quality of life (National Eye Institute Visual Function Questionnaire [NEI-VFQ]-25 and EuroQol 5-Dimension 5-level [EQ-5D-5L]) and patient-reported outcomes measurements (CatQuest-9SF) preoperatively and 3 months postoperatively in the appropriate language.^14–16^

### Statistical Analysis

Statistical analyses were performed using IBM SPSS Statistics for Windows v. 29.0 (IBM Corp.). All quantitative variables were summarized. The data were tested for normal distribution, and the appropriate (non-)parametric test was used. For all analyses, a *P* ≤ .05 is considered statistically significant. Comparisons between the web-based assessments and the conventional clinical assessments were analyzed with paired *t* tests and in line with the Bland-Altman methodology.^17^ Mean differences (ie, bias) and 95% limits of agreement (LoA) (ie, sampling error) were presented. Residual refractive error outcomes were converted to absolute values of the SEQ. Associations between clinical characteristics and accuracy of the web-based test were evaluated by generalized estimating equation (GEE) analysis correcting for bilaterality, age, sex, myopic target refraction, and self-reported vision symptoms. Scores of the self-reported outcome measurements were calculated using official scoring manuals and conversion sheets. Differences between the 2 follow-up groups regarding these outcomes were evaluated with an independent samples *t* test.

## RESULTS

### Recruitment

A total of 391 patients were invited to participate. The participant recruitment is depicted in Supplemental Figure 1 (available at http://links.lww.com/JRS/B155). The willingness and ability of invited patients to enter the study varied between countries: 41% in the Netherlands, 14% in Germany, and 9% in Austria. There was no difference in age between those who were willing and able to participate and those who were not (70 ± 7 vs 71 ± 11, *P* < .001).

A total of 94 participants (188 eyes) were enrolled at baseline. Table 1 presents their baseline characteristics.

**Table 1.** Baseline characteristics

Clinical characteristics	Telemonitoring group (n = 44)	Usual care group (n = 50)
Age (y), mean (SD) [range]	70 (6) [54, 80]	70 (8) [47, 87]
Sex, n (%)		
M	23 (52)	23 (46)
F	21 (48)	27 (54)
Nationality, n (%)		
Dutch	34 (77)	38 (76)
German	3 (7)	3 (6)
Austrian	7 (16)	9 (18)
Preop presenting VA score at the clinic (in logMAR), mean (SD)^a^	0.28 (0.17)	0.34 (0.26)
Preop refractive error (SEQ in D), mean (SD)	−1.22 (2.97)	−1.43 (3.99)
History of eye diseases and/or eye surgeries, n (%)		
Refractive surgery	3 (7)	0 (0)
Blepharoplasty	2 (5)	4 (8)
Strabismus surgery	1 (2)	1 (2)
Post target refraction, n (%)^b^		
Emmetropia	37 (84)	40 (80)
Myopia (SEQ <−0.5 D)	7 (16)	10 (20)
Surgical complicating factors, n (%)^c^		
None	40 (91)	50 (100)
Corneal clouding	0 (0)	0 (0)
Small pupil	1 (0.02)	0 (0)
Mature cataract	0 (0)	0 (0)
Pseudoexfoliation syndrome	1 (0.02)	0 (0)
Other (eg, shallow anterior chamber)	2 (0.05)	0 (0)
CatQuest-9SF score (scale −6.00 to 6.00), mean (SD)	3.03 (0.43)	3.06 (0.62)
NEI-VFQ-25 composite score (scale 0-100), mean (SD)	75 (12)	74 (15)
General health condition: self-rated health score at baseline (scale 0%-100%), mean (SD)	83 (12)	82 (15)
EQ-5D-5L Index Values (scale 0.00-1.00), mean (SD)	0.96 (0.06)	0.91 (0.13)

EQ-5D-5L = EuroQol 5-Dimension 5-level; NEI-VFQ-25 = National Eye Institute Visual Function Questionnaire-25; SEQ = spherical equivalent

a Preoperative VA was assessed with current prescription, if applicable

b Target refraction was similar for both eyes

c Potentially complicated surgical cases where not explicitly excluded from participation

### Accuracy of Web-Based Vision Self-Assessment (Telemonitoring Group Only)

#### Comparison of Web-Based vs Conventional VA Assessment

**Figure 3.**
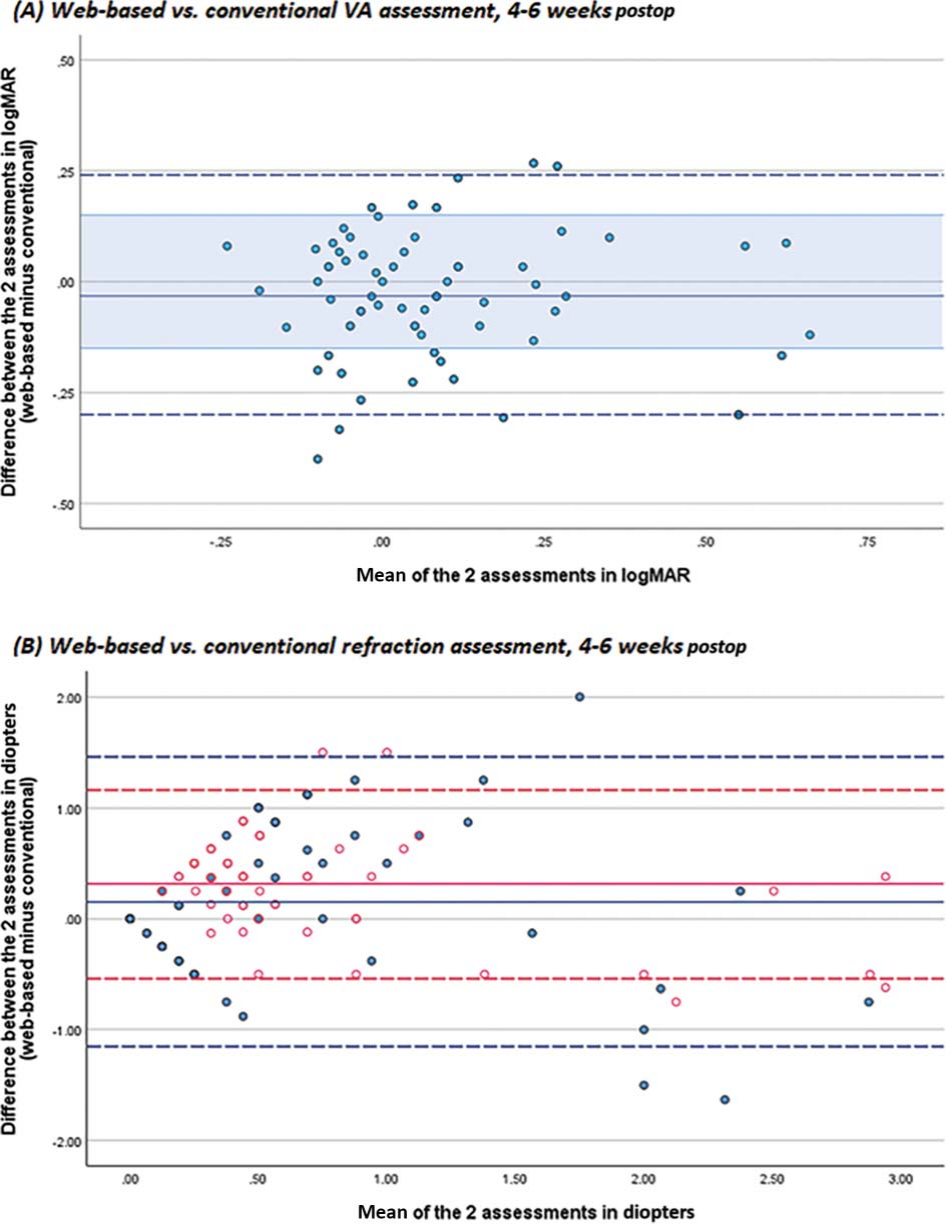
Comparisons of web-based vs conventional assessments (telemonitoring participants only). Please note that these 2 assessments were compared within the same subject and, therefore, only refer to the telemonitoring group. The *straight lines* represents the mean difference; the *dashed lines* the 95% limits of agreement. The semitransparent blue zone in *A* depicts the predetermined clinically acceptable deviation range of ±0.15 logMAR. Note that an update of the web-based refraction algorithm had taken place after the trial, resulting in a new method of SEQ calculations. The *blue lines* and *blue circles* in *B* represent the original web-based refraction algorithm; the *red lines* and *red circles* represent the updated algorithm. SEQ = spherical equivalent

The graphical comparison between the VA assessments at home and at the clinic at 4 to 6 weeks postoperatively is depicted in Figure 3, A. These comparisons were made within the same subject and only refer to the telemonitoring group. The mean difference was −0.03 ± 0.14 logMAR (*P* = .07), indicating that there is no systematic under or overestimation of the VA by the web-based tool. The 95% LoA ranged from −0.30 to 0.24 logMAR, and most assessments fall within the predetermined acceptable range of ±0.15 logMAR. Repeatability of the VA assessment showed a fairly similar precision: a mean difference of 0.02 ± 0.14 logMAR (*P* = .15), with 95% LoA ranging from −0.25 to 0.29 logMAR (1 vs 3 months postoperatively, see Supplemental Figure 2, available at http://links.lww.com/JRS/B156). Six participants were excluded from this repeatability comparison as they had reported a change in vision over the 2-month interval. GEE analysis revealed no associations between any of the examined clinical variables and accuracy of the web-based VA assessment (see Supplemental Table 1, available at http://links.lww.com/JRS/B157).

#### Comparison of Web-Based Refraction vs Manifest Refraction at the Clinic

A graphical comparison between the web-based and subjective refraction outcomes at 4 to 6 weeks postoperatively is depicted in Figure 3, B. These comparisons were made within the same subject and only refer to the telemonitoring group. The mean difference in SEQ was 0.15 ± 0.67 D (*P* = .07), with 95% LoA ranging from 1.15 to 1.46 D. The updated algorithm generated a reduced variability, yet with a higher and significant systematic bias: a mean difference of 0.32 ± 0.43 D (*P* < .01), with 95% LoA ranging from −0.54 to 1.16 D. GEE analysis revealed that age and the presence of self-reported symptoms in the triage questionnaire before the web-based test are significantly associated with the accuracy of the SEQ assessment, although the extent of these effects (β = 0.01 and β = 0.17) are not considered clinically relevant (see Supplemental Table 1, available at http://links.lww.com/JRS/B157).

The data demonstrate that the algorithm tends to overestimate the absolute residual refractive error. The web-based test's ability to determine a residual refractive error >0.5 D has a sensitivity of 88% and specificity of 67%, meaning that there will be a substantial number of false positives (ie, patients with emmetropia will falsely be classified as ametropic). The attained mean CDVA achieved with the algorithm's web-based refraction increased compared with the mean preoperative CDVA (0.1 vs 0.3 logMAR), yet was inferior to the mean CDVA achieved with the prescription of the subjective refraction assessed at the clinic: 0.1 vs −0.1 logMAR (ie, 0.8 vs 1.2 Snellen decimal). An improvement of 1 or more lines from the preoperative VA was achieved for 63% of the eyes using the web-based refraction and for 96% of the eyes using the manifest refraction.

#### Human Over-read

An unacceptable high within-test variation (ie, during the same monocular examination) was identified for 15 assessments (in 13 patients); these were excluded from the overall analyses. This subgroup was not evidently different at baseline in terms of age (71 ± 5 years) or sex (46% female) when compared with other telemonitoring participants. Interestingly, 7 of these eyes had a myopic target refraction (ie, half of all myopic eyes included in the trial) and 1 eye was aphakic because of an interoperative complication; underlining that a poor uncorrected distance VA is associated with a high within-test variation. GEE analysis confirmed this with a positive association between a myopic target refraction and a high within-test variation. No other clinical parameters were identified in this aspect (see Supplemental Table 1, available at http://links.lww.com/JRS/B157).

### Safety

#### Adverse Events and Postoperative Management Changes

Most of the participants did not have a face-to-face consultation in the first week postoperatively: Only 18 participants visited the clinic (36% of the usual care group). These visits had been scheduled preoperatively in line with the clinic's usual practice and were all uneventful.

In addition to the scheduled consultations, 12 usual care and 7 telemonitoring participants contacted or visited the clinic (24% vs 16% of each group total) (as defined in Figure 1). These unexpected consultations and consecutive management changes are presented in Table 2. Three surgical complications were registered: 1 residual cortical lens matter in the anterior chamber, 1 wrong lens implantation, and 1 eye was left aphakic because of intraoperative difficulties. All of these underwent successful reoperations.

**Table 2.** Reasons for unexpected consultations (independent of the study scheme)

Reason for consultation	Usual care participants (n = 12)	Telemonitoring participants (n = 7)	Management change
No pathology, n (%)	3 (25)	4 (57)	Reassurance of the patient
Dry-eye syndrome or blepharitis, n (%)	5 (42)	1 (14)	Addition of lubricating drops
Postoperative inflammation, n (%)	2 (17)	1 (14)	Addition of anti-inflammatory drops
Intraoperative complication, n (%)	2 (17)	1 (14)	Reoperation

#### Triage Questionnaires

In addition to the web-based eye tests, the telemonitoring participants were asked to fill out triage questionnaires. The reported symptoms are presented in Table 3.

**Table 3.** Outcomes of self-reported triage questionnaires

Self-reported symptom in triage questionnaire, n (%)	<1 wk postop Total n = 87 eyes^a^	±1 mo postop Total n = 85 eyes^a,b^
None	33 (38)	41 (48)
Photophobia	25 (29)	18 (21)
Itchiness	7 (8)	9 (11)
Oppressive feeling	4 (5)	7 (8)
Gritty feeling	4 (5)	11 (13)
Burning sensation	3 (3)	7 (8)
Pain	3 (3), mean score = 2/10	4 (5), mean score = 2/10
Free text: “tears”	0 (0)	4 (5)
Free text: “dry eyes”	0 (0)	3 (4)
No response to the sent questionnaire	10 (11)	4 (5)

a Surgery cancelled for 1 eye

b One study drop-out postoperatively (both eyes)

### Self-Reported Outcome Measurements

The response rate of the questionnaires at baseline and 3 months postoperatively was 100%. An overview of the self-reported outcome measurements is presented in the Supplemental Table 2 (available at http://links.lww.com/JRS/B158). Visual functioning (indicated by the Catquest-9SF and NEI-VFQ-25 scores) improved postoperatively for all participants (with a mean improvement of −0.80 and 16.70, respectively), and performing vision self-assessments did not materially affect these scores, as there were no significant differences between the 2 follow-up groups. The effects of cataract surgery on EQ-5D-5L index scores were marginal in both groups.

## DISCUSSION

Our study evaluated remote cataract surgery follow-up involving self-assessments of visual function and self-reported outcome measurements using telehealth technology. The studied web-based postoperative VA assessment, performed independently at home, showed a mean difference of 0.03 ± 0.14 logMAR when compared with conventional ETDRS testing. This negligible mean difference indicates that there is no fixed bias, meaning that the web-based test does not systematically over or underestimate VA. The 95% LoA (ie, the distribution of differences) ranged from −0.30 to 0.24 logMAR, indicating that outlier assessments occurred beyond the predetermined clinical deviation limit of ±0.15 logMAR. The web-based refraction algorithm resulted in an overestimation of the residual refractive error and will falsely indicate a residual refractive error in patients with emmetropia. Adverse events were rare and occurred in both groups. Telemonitoring and self-reporting led to a more detailed overview of postoperative symptoms and complaints while unscheduled consultations were recorded less often. We acknowledge that the study was not powered on adverse events and postoperative management changes. Vision-related quality of life improved postoperatively with no apparent differences between groups.

The mean difference between the web-based VA assessment and the conventional chart was clinically negligible. However, when evaluating agreement, it is crucial to look into the distribution of differences. It is important to realize that a certain degree of variation is unavoidable when comparing different charts, given the diversity in optotypes and scoring criteria.^11,18^ An authoritative study comparing repeated VA assessments with various charts and observers concluded that differences exceeding ±0.15 logMAR should, therefore, be considered clinically relevant.^11^ In our study, we identified outcomes beyond this range. It should be noted that the occurrence of variation in the absence of clinical changes is a common feature of VA testing, attributed to behavioral factors such as fatigue or intrinsic motivation. This has been demonstrated by repeatability (ie, test-retest studies) that evaluate repeated VA assessments using the same chart in the same person. For the ETDRS chart, repeatability studies have shown 95% LoA ranging up to ±0.18 logMAR.^12,19^ For the Snellen chart, the variability is higher, with 95% LoA ranging up to ±0.24 logMAR using the single-letter method and up to ±0.33 logMAR using the line assignment method (ie, the test is terminated when half of the letters on the line are misread).^12,19^ The wide distribution of measurement variability indicated by the 95% LoA underlines that the occurrence of outlier measurements is an inherent aspect of assessing VA. Notwithstanding, the here-found agreement is subpar to the precision reported in ETDRS test-retest studies, prompting future efforts to increase the measurement precision of the web-based test.

Multiple telehealth tools for self-assessing visual function are available on the internet, although many have not been validated.^20^ Those that are validated have mostly been evaluated in controlled settings, prone to observer bias.^19^ A previous study evaluated the performance of the here-studied web-based test in a supervised hospital setting and reported a slightly better precision regarding the uncorrected VA assessment after cataract surgery (95% LoA −0.26 to 0.15 logMAR).^21^ Unsupervised home assessments will arguably be more affected by behavioral and environmental factors than assessments in controlled settings. In this study, the web-based test was performed independently by patients at home, reflecting a real-world scenario.

Future software updates should be aimed at improving the measurement precision of the web-based test. This involves new technical integrations, for instance, by using webcam images and providing AI-guided live feedback to optimize testing conditions at home (eg, considering lighting conditions and distance to the screen). Moreover, the ease by which repeated measurements can be performed is an interesting feature of home-based self-assessments, potentially reducing the variability of VA results. The web-based test could benefit from automated detection of within-test inconsistencies indicative of poor performance, with the subsequent functionality to let patients repeat a specific part of the test or to redo the complete test at a later point in time under optimal testing conditions.

It is important to address the suboptimal performance of the web-based refraction assessment in this study population. While an improvement in VA is indicative of surgical success, most cataract guidelines also recommend a refraction assessment postoperatively.^22^ Our findings indicate that the current version of the web-based refraction assessment is not able to reliably determine refractive error in this patient population. The current version has a tendency to falsely identify a residual refractive error in patients with emmetropia. This is an important limitation. The algorithm, therefore, requires a recalibration before it can be useful to collect surgery outcomes for mandatory registries. It was trained on a population of young adults (aged 18 to 40 years) with an excellent VA (>1.25 Snellen decimal).^23^ VA has been reported to decrease with age, even in healthy eyes, particularly after 55 years of age.^24^ The assumption in the algorithm that any VA poorer than −0.1 logMAR requires a refractive correction will, therefore, by design result in an overestimation of the residual refractive error in older adults. A data set comprising normative best corrected VA scores after uneventful post-cataract surgery (based on 490.240 records) has been obtained from the European Registry of Quality Outcomes for Cataract and Refractive Surgery and will be used to recalibrate the algorithm. An updated and accurate self-assessment of residual refractive errors postoperatively would be an important step for automated data collection and quality registration.

The quality of vision as perceived by the patient may be influenced by parameters other than VA. Patient-reported data on vision-related quality of life have become important indicators for surgical success.^25^ These parameters are often under-reported in conventional care. Interestingly, the response rate of these questionnaires in this study was 100%. We believe that collection of self-reported data relies on adequate instructions, a perceived benefit of registration, and a good and accessible digital infrastructure. Therefore, engaging patients in their eye health by self-monitoring their vision might build on this perception of usefulness and lower thresholds to fill out these outcome-related questionnaires. In addition, a well-integrated web-based platform could be of great value for maintaining feedback loops to cataract surgeons, by collecting data that are relevant for quality control and benchmarking.

Public support is of paramount importance for successful adoption of a remote follow-up practice. Some eyecare professionals might be reluctant to adopt remote care practices as they fear vision-threatening postoperative complications. Notwithstanding, a substantial body of clinical experience and scientific evidence has demonstrated the safety and efficacy of telephone follow-up alternative to face-to-face visits.^5,26^ Unexpected management changes are unlikely in asymptomatic patients after uncomplicated surgery, and a structured set of clinical questions (such as pain, redness, and decreased vision) has been proven sufficient to risk-stratify patients.^22,27^ A recent innovation is an automated telephone consultation with AI-driven clinical assistants, combining a low-tech method of delivery with a high-tech *large language model*–driven solution.^28^ A potentially more accessible and scalable alternative could be a self-administered digital questionnaire, as in this study, or a combination of both: an interactive questionnaire providing personalized feedback based on the responses. Patients could, for instance, be reminded to apply their lubricating drops as most reported symptoms in the triage questionnaires relate to dry eyes. An automated alert system, integrated with the clinic's electronic health record should identify those in need of a follow-up consultation.

Finally, we would like to underline that conventional follow-up care involving face-to-face visits should always remain accessible. Although high patient satisfaction rates for remote cataract surgery follow-up, involving (automated) telephone consultations and/or self-assessments of visual function, have been reported, the recruitment phase of this trial seemed challenging.^29–31^ Regional differences in the ability and willingness to participate were identified, with participation rates being highest in the Netherlands. This was mainly rooted in technology adoption barriers. In Germany and Austria, a notable proportion of invited patients could not participate because of a lack of access to mobile devices or insufficient proficiency to perform self-assessments at home. The participant rates align with Eurostats data indicating that the Netherlands surpasses both Germany and Austria by having the highest levels of internet usage and greatest number of citizens possessing basic digital skills in Europe.^32^ Internet access has, however, been growing rapidly across Europe over the past decade, and we expect the group of digitally skilled patients with cataract to grow exponentially in the upcoming years. In reality, a group of patients will remain not suitable for remote follow-up. Preoperative counselling and identifying those at risk of complications and postoperative management changes is crucial. This includes patients with known risk factors of endophthalmitis (eg, blepharitis, ectropion), uncontrolled intraocular pressure elevation (eg, glaucoma, ocular hypertension), or postoperative inflammation (ie, uveitis); those who lack the (digital) skills to self-report their own clinical data; or those who are not willing to do so.^33–36^ In addition, even when the patient was initially planned for remote follow-up, thresholds for call-backs to the clinic should be low.

In conclusion, our study showed that a group of digitally skilled patients with cataract were able to complete unsupervised self-assessments of visual function and self-report postoperative outcome measures. This underlines the potential of telemonitoring after cataract surgery. Adverse events or (vision-related) quality of life did not differ between the 2 follow-up groups. Future adjustments should aim to further improve the precision of the web-based test. The biggest challenges revolve around the inevitable occurrence of outlier assessments, which could be more frequent when vision assessments are performed unsupervised by patients at home. Software updates of the web-based tool should be aimed at identifying and limiting these outliers, and design automated feedback loops that invite to retest when an outlier is suspected. The current version of the web-based refraction assessment does not deliver reliable outcomes in this population and requires a recalibration, with future training building on a normative data set derived from the European Registry of Quality Outcomes for Cataract and Refractive Surgery. We believe that remote self-care can be a promising avenue to comply with increasing demands of cataract care, and improvements of technology and public support could make web-based self-collection of postoperative outcome data become a reality.

**WHAT WAS KNOWN**

- Demands for cataract surgery are ever-increasing. Although national protocols vary, conventional cataract surgery follow-up typically consists of routine face-to-face examinations to assess postoperative visual function and detect adverse events
- The high volume of surgical procedures and low adverse event rates make routine cataract surgery follow-up an interesting field for using telehealth for remote care delivery.
- A web-based platform for collecting self-assessments of visual function and self-reported clinical outcome measures has yet to be evaluated.

**WHAT THIS PAPER ADDS**

- Digitally skilled patients with cataract are able to complete unsupervised self-assessments of visual function and self-report clinical outcome measures using a web-based platform.
- Both conventional and web-based follow-ups yielded similar patient-reported outcome measurements outcomes and adverse event rates, suggesting less unscheduled clinical visits with web-based follow-ups.
- Future adjustments should aim to further improve the precision of the web-based test. The biggest challenges revolve around the inevitable occurrence of outlier assessments, which could be more frequent when vision assessments are performed unsupervised by patients at home.
